# CT staging performance in an international trial of neoadjuvant chemotherapy for locally advanced colon cancer

**DOI:** 10.1093/bjr/tqaf217

**Published:** 2025-08-23

**Authors:** James R Platt, Faye Elliott, Kelly Handley, Laura Magill, Philip Quirke, Matthew T Seymour, Nicholas P West, Dion Morton, Jenny Seligmann, Damian J M Tolan

**Affiliations:** Division of Oncology, Leeds Institute of Medical Research at St. James’s, School of Medicine, University of Leeds, Leeds, LS2 9JT, United Kingdom; Division of Haematology and Immunology, Leeds Institute of Medical Research at St. James’s, School of Medicine, University of Leeds, Leeds, LS2 9JT, United Kingdom; Birmingham Clinical Trials Unit (BCTU), College of Medicine and Health, University of Birmingham, Birmingham, B15 2TT, United Kingdom; Birmingham Clinical Trials Unit (BCTU), College of Medicine and Health, University of Birmingham, Birmingham, B15 2TT, United Kingdom; Division of Pathology & Data Analytics, Leeds Institute of Medical Research at St. James’s, School of Medicine, University of Leeds, Leeds, LS2 9JT, United Kingdom; Division of Oncology, Leeds Institute of Medical Research at St. James’s, School of Medicine, University of Leeds, Leeds, LS2 9JT, United Kingdom; Division of Pathology & Data Analytics, Leeds Institute of Medical Research at St. James’s, School of Medicine, University of Leeds, Leeds, LS2 9JT, United Kingdom; Institute of Applied Health Research, College of Medicine and Health, University of Birmingham, Birmingham, B15 2TT, United Kingdom; Division of Oncology, Leeds Institute of Medical Research at St. James’s, School of Medicine, University of Leeds, Leeds, LS2 9JT, United Kingdom; Department of Radiology, Leeds Teaching Hospitals NHS Trust, Leeds, LS9 7TF, United Kingdom

**Keywords:** colon cancer, neoadjuvant, chemotherapy, clinical trial, staging, computed tomography, radiology, pathology

## Abstract

**Objectives:**

In the Fluorouracil, Oxaliplatin and Targeted Receptor pre-Operative Therapy (FOxTROT) trial, neoadjuvant chemotherapy (NAC) significantly reduced recurrence risk, compared to upfront surgery, in locally advanced colon cancer. This analysis evaluates the correlation between radiological and pathological staging within the trial to support the adoption of CT-based patient selection.

**Methods:**

In this preplanned analysis of prospectively collected data, local radiological and pathological staging were compared in upfront surgery participants. T stage, N stage, and extramural venous invasion (EMVI) status were evaluated using overall agreement, sensitivity, specificity, positive predictive value (PPV) and negative predictive value (NPV). Subgroup analyses explored the impact of mismatch repair status and tumour side.

**Results:**

A total of 354 participants were included. T stage agreement was 63.0%; T3 and T4 tumours were correctly identified in 78.9% and 41.1% of participants, respectively. The PPV for T3-4 status was 94.5%. N stage agreement was 39.8%; for N status (positive vs. negative), overall agreement, sensitivity, specificity, PPV, and NPV were 54.1%, 81.1%, 26.0%, 53.2%, and 57.1%, respectively. For EMVI, these values were 54.9%, 71.0%, 41.2%, 50.7%, and 62.5%, respectively. Accuracy metrics did not differ significantly by tumour side or mismatch repair status.

**Conclusions:**

CT effectively predicted T3-4 status with minimal overstaging, but performed poorly for individual T stage, N stage, and EMVI. We propose radiological T3-4 status should be adopted as the primary biomarker for neoadjuvant patient selection, with molecular biomarkers to guide treatment choice.

**Advances in knowledge:**

In this multicentre trial, local radiologists accurately identified T3-4 status to select participants for NAC, indicating utility for future neoadjuvant trials and clinical practice.

## Introduction

The international Fluorouracil, Oxaliplatin and Targeted Receptor pre-Operative Therapy (FOxTROT; NCT00647530) study was the first phase III, randomized-controlled trial to evaluate neoadjuvant chemotherapy (NAC) in locally advanced but resectable colon cancer (CC).^1^^,^^2^

In contrast to adjuvant chemotherapy, where pathological staging guides treatment decisions, patient selection for NAC is based upon radiological (clinical) tumour, node, metastasis (TNM) staging using CT.^1^^,^^3^ FOxTROT selected patients with radiological T3-4 N0-2 M0 staging and determined that 6 weeks of NAC significantly reduced the risk of recurrence at 2 years, compared to immediate surgery.^1^ Furthermore, NAC was associated with lower pathological T and N stages, the absence of extramural venous invasion (EMVI) and lower rates of incomplete surgical resection and postoperative complications. Therefore, NAC can be considered a safe and effective treatment for locally advanced CC.

The limitations of matching radiological and pathological staging are well-recognized and the selection of patients with CC for NAC using CT represents a major shift in practice.^4^^,^^5^ In addition, the recent emergence of neoadjuvant immunotherapy for patients with mismatch repair (MMR) deficient (dMMR) CC indicates radiological patient stratification will become increasingly important.^6^ CT is the gold standard investigation for radiological evaluation with similar diagnostic accuracy compared to MRI and PET-CT.^7^ However, as the neoadjuvant treatment landscape evolves, alternatives to CT may provide effective imaging biomarkers for patient selection and treatment-response assessment. While radiological staging performance in CC has been comprehensively evaluated, some studies are limited by small sample size, retrospective data, or single centre design, and others present data where staging assessments are unlikely to have been performed with patient selection for NAC in mind.^4^^,^^5^^,^^8–17^ Understanding the correlation between radiological and pathological assessment of T stage, N stage, and EMVI status within a multicentre trial context could accelerate moving CT stratification for personalized CC neoadjuvant therapies into routine clinical practice.

## Methods

The FOxTROT study protocol was granted ethical approval (MREC: 07/S0703/57), including analysis of prospectively collected CT scan data, with all participants providing written consent. The main trial results have already been published.^1^

### Study design

A preplanned analysis to correlate radiological and pathological staging was performed in participants randomized to the straight-to-surgery (STS) arm, who therefore had not received NAC. Participants randomized to the NAC arm were not included in this analysis due to the downstaging effect of NAC, following which pre-treatment radiological and post-treatment pathological staging are not directly comparable.

Participants had radiologically staged T3 (with ≥1 mm extension beyond the muscularis propria) or T4 non-metastatic CC on baseline CT. While the degree of extension beyond the muscularis propria, N stage, and EMVI status were also predicted and recorded by local site radiologists, they were not considered for trial eligibility. Patients with bowel obstruction were eligible, providing the obstruction was first relieved with a defunctioning stoma. Eligible patients were identified during local multidisciplinary team (MDT) meetings across 85 sites in the United Kingdom, Denmark, and Sweden between May 15, 2008 and December 23, 2016.

### Pathological staging

The reference standard was pathological staging of the surgical specimen, according to TNM version 5, which was recorded on a detailed pathology proforma supported by a written protocol.^18^ A lead pathologist at each trial site attended in-depth face-to-face pathology training. Pathologists were not specifically blinded to results from pre-treatment scans.

### Radiological staging

Radiological staging was the index test, which was performed at baseline using a portal venous phase, contrast-enhanced, spiral/multidetector CT of the thorax, abdomen, and pelvis with maximum 5 mm slice thickness. Oral contrast was permitted according to radiologist preference. A lead radiologist at each trial site attended in-depth face-to-face radiology training (led by a specialist gastrointestinal radiologist with over 10 years’ experience) on image interpretation and tumour analysis to guide reporting using TNM version 5.^18^ At least 148 radiologists (unnamed for 115/1052 [10.9%] CT scans) from 85 sites reported CT scans in the trial. Initial MDT review of radiological staging was used to confirm trial eligibility (as reported in the main trial publication).^1^ A subsequent detailed review of the tumour was provided by the trained lead radiologist; differences between these reviews are detailed in the Supplementary Data. The local lead radiologist review is considered the final local centre radiology opinion for clinical staging for this analysis and subsequent publications for this trial.

### Data collection

Local radiological staging data were submitted by the lead radiologist using a detailed case report form. Local pathological staging data were also submitted using a case report form. MMR status was retrospectively determined using immunohistochemistry for MLH1, PMS2, MSH2, and MSH6 at the central trial laboratory on either resection or biopsy specimens.

### Statistical analysis

T stage, N stage, and EMVI status were assessed for radiological and pathological agreement. T stage and N stage were further evaluated as dichotomous outcomes known as T status (T1-2 vs. T3-4) and N status (N0 vs. N1-2[+]), which are more clinically relevant for treatment decisions. Staging subgroups (e.g. T4a vs. T4b) were not considered. However, since other studies have defined T3 with ≥5 mm extramural extension as a high-risk group of interest, we performed a further assessment of T status using this threshold.^19^

Staging assessments were evaluated against MMR status and primary tumour side (right vs. left colon), which have been reported to influence radiological staging accuracy.^20^^,^^21^ For this analysis, right-sided tumours were defined as those proximal to the splenic flexure and left-sided tumours those at, or distal to, the splenic flexure.

Overall agreement was calculated for each staging outcome. The sensitivity, specificity, positive predictive value (PPV), and negative predictive value (NPV) of CT were calculated for each dichotomous outcome (T status limited to PPV only) and reported with 95% CIs. Overall agreement was calculated as the number of participants with concordant radiological and pathological staging divided by the total number of participants with complete data. The other metrics were calculated according to standard definitions.^22^ The 95% CIs for overall agreement were calculated using MedCalc (MedCalc Software Ltd. Diagnostic test evaluation calculator Version 22.023, Ostend, Belgium). STATA software was used for all other analyses (StataCorp, Stata Statistical Software: Release 16, College Station, TX, United States: StataCorp LLC). Illustrations were prepared in Microsoft Powerpoint (Microsoft, Version 16.97, Redmond, WA, United States) and Python (Python Software Foundation, Version 3.11.5, Wilmington, DE, United States) using the Matplotlib (Version 3.7.2) and Seaborn (Version 0.12.2) libraries.

## Results

### Participant characteristics

**Figure 1.**
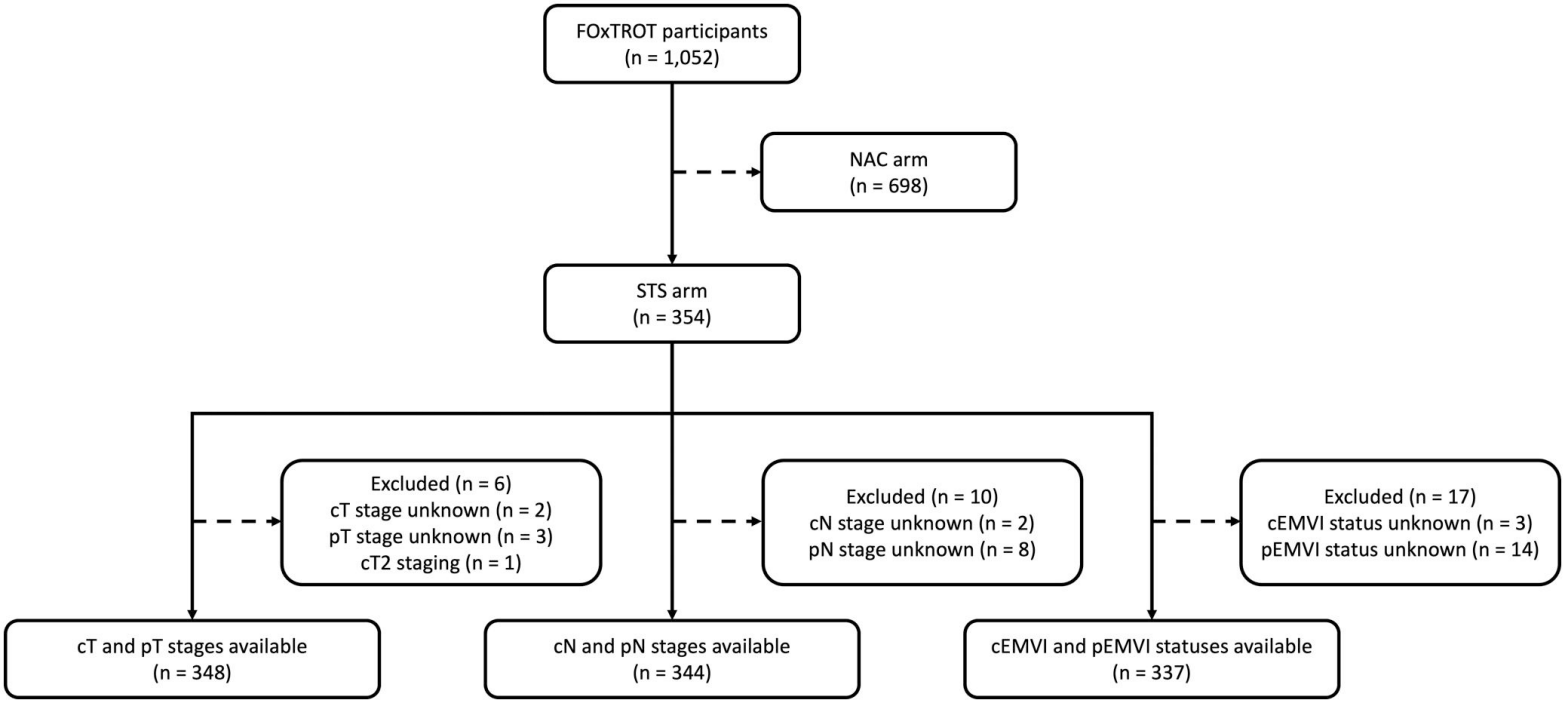
Selection of participants. Assessments of radiological and pathological correlation were performed for participants in the STS arm. For each part of the analysis, participants were excluded if either of the corresponding radiological or pathological data were unknown. Abbreviations: c = radiological; EMVI = extramural venous invasion; N = lymph node; NAC = neoadjuvant chemotherapy; p = pathological; STS = straight-to-surgery; T = primary tumour.

A total of 354 participants were randomized to the STS arm and therefore included in this analysis, comprising 225 men and 129 women (mean age 63.2 years). Baseline clinical and staging characteristics for trial participants have been reported previously (Table S1).^1^ Some differences were observed for tumour location, T stage, N stage, and extramural extension between initial MDT review and trained lead radiologist review, but crucially, only 2/1052 (0.2%) were revised from T3 to T2 staging (Table S2). Figure 1 illustrates the selection of participants for each component of the analysis.

### T staging

Both radiological and pathological T stage data were available in 98.5% (349/354) of participants (Figure 1).

**Figure 2.**
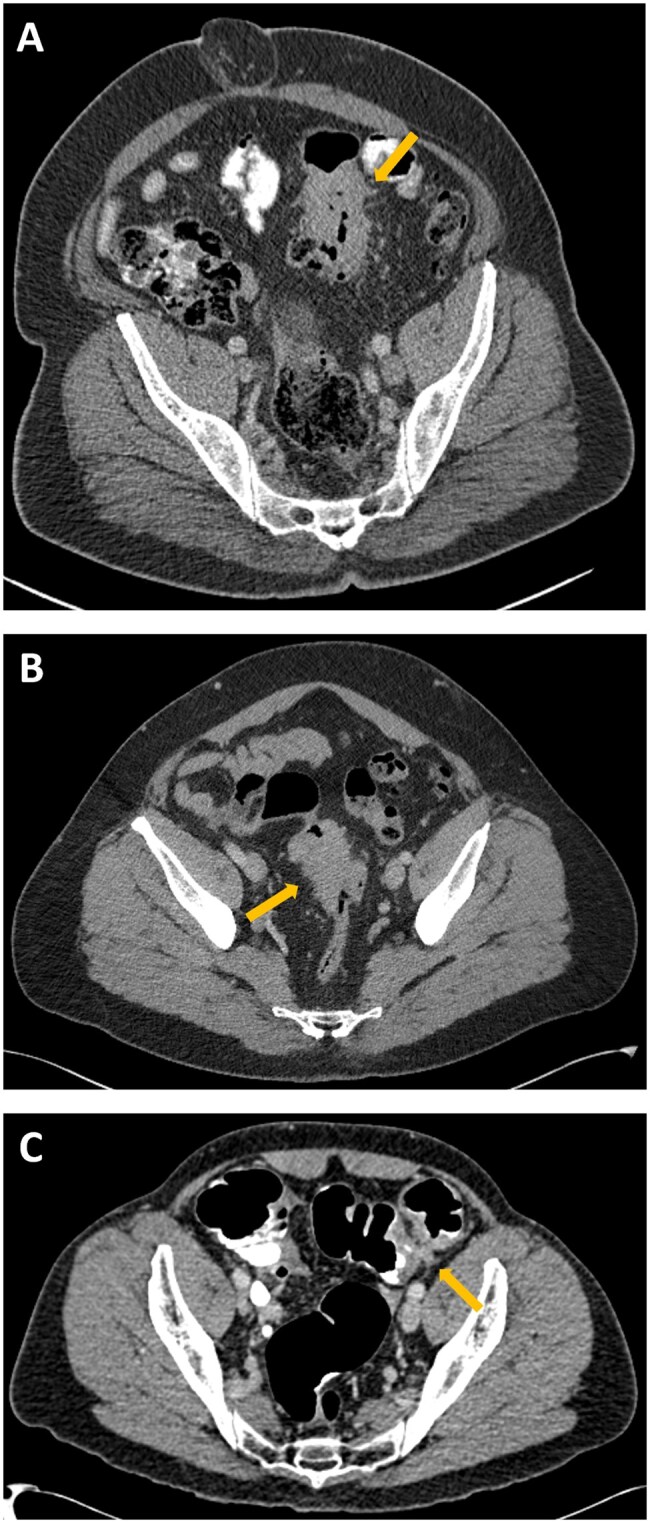
Example T staging. Baseline axial images of participants in the straight-to-surgery arm. (A) Correctly staged T3 sigmoid tumour. Yellow arrow indicates area of extension beyond the muscularis propria. (B) pT2 sigmoid tumour that was over-staged as cT3. Yellow arrow indicates the primary tumour. (C) pT3 sigmoid tumour that was over-staged as cT4 with predicted involvement of peritoneum. Yellow arrow indicates area of suspected peritoneal invasion.

**Figure 3.**
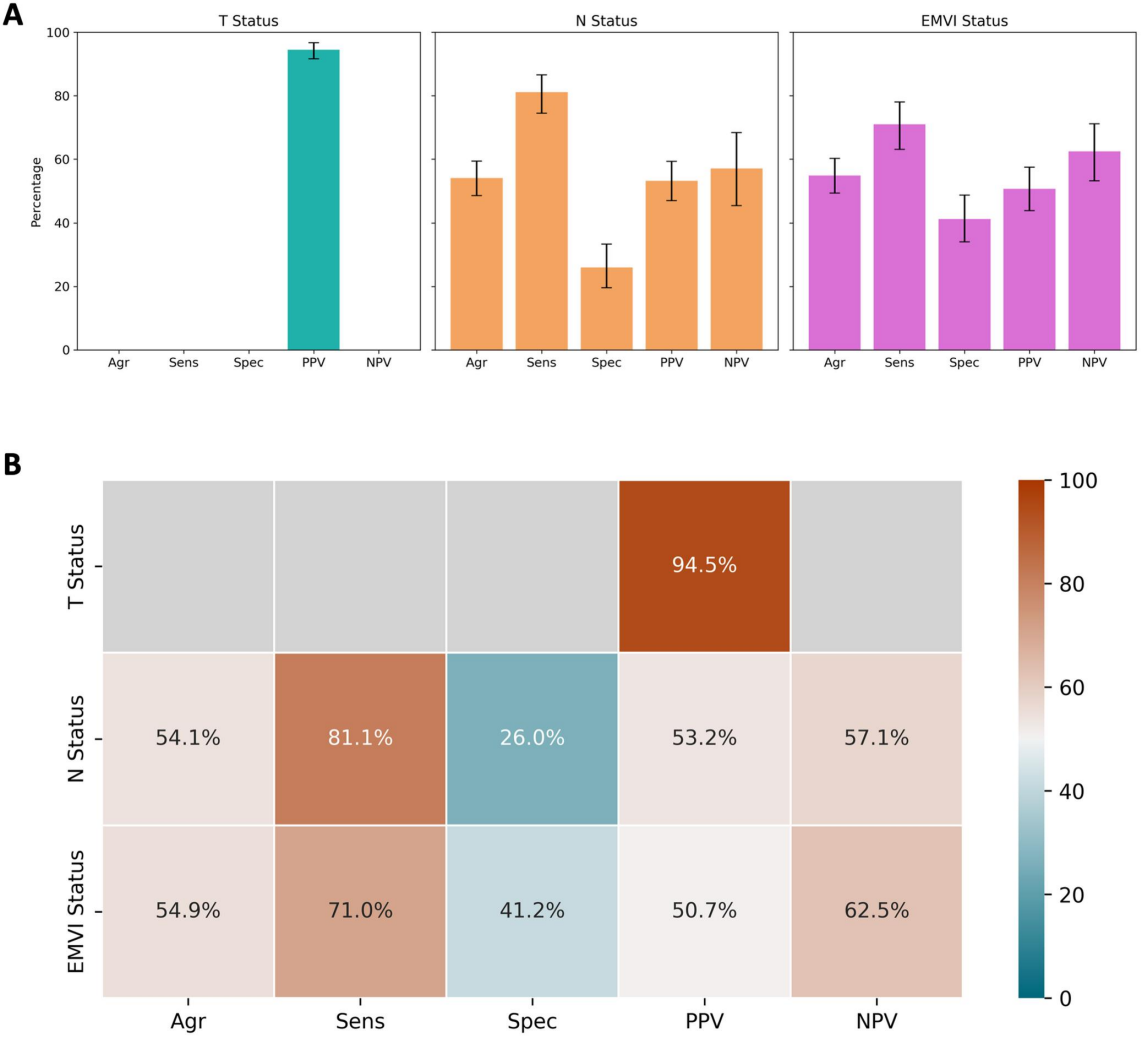
Radiological staging performance in all participants. The overall agreement, sensitivity, specificity, PPV and NPV of CT for predicting T, N, and EMVI statuses shown as (A) individual bar charts and (B) a combined heatmap. Only PPV could be calculated for T status due to the exclusion of earlier stages of disease from the trial. Error bars represent 95% CIs. Abbreviations: Agr = overall agreement; EMVI = extramural venous invasion; N = lymph node; NPV = negative predictive value; PPV = positive predictive value; Sens = sensitivity; Spec = specificity; T = primary tumour.

The overall agreement for T stage was 63.0% (220/349), with T3 and T4 tumours correctly identified in 78.9% (176/223) and 41.1% (44/107) of participants, respectively (Table 1; Figure 2). Calculation of the sensitivity, specificity, and NPV of CT for predicting T3-4 status was not possible due to the exclusion of patients radiologically staged T1-2 from the trial. However, the PPV for predicting T3-4 status was 94.5% (329/348) (Tables 1 and 2; Figure 3). With the T status threshold adjusted to T3 (≥5 mm extramural extension)-T4 staging, the PPV was 68.7% (173/252) (Table S3). Of the 20 participants with pathologically staged T1-2 tumours, 8 had an alternative high-risk pathological feature (N+ or EMVI).

**Table 1.** Radiological and pathological correlation for T stage, N stage, and EMVI status.

	pT1	pT2	pT3	pT4	Total
cT1	0 (0.0)	0 (0.0)	0 (0.0)	0 (0.0)	0 (0.0)
cT2	0 (0.0)	0 (0.0)	0 (0.0)	1 (0.3)	1 (0.3)
cT3	1 (0.3)	15 (4.3)	176 (50.4)	62 (17.8)	254 (72.8)
cT4	0 (0.0)	3 (0.9)	47 (13.5)	44 (12.6)	94 (26.9)
Total	1 (0.3)	18 (5.2)	223 (63.9)	107 (30.7)	349

	pT1-2	pT3-4	Total
cT1-2	0 (0.0)	1 (0.3)	1 (0.3)
cT3-4	19 (5.4)	329 (94.3)	348 (99.7)
Total	19 (5.4)	330 (94.6)	349

	pN0	pN1	pN2	Total
cN0	44 (12.8)	19 (5.5)	14 (4.1)	77 (22.4)
cN1	82 (23.8)	51 (14.8)	34 (9.9)	167 (48.5)
cN2	43 (12.5)	15 (4.4)	42 (12.2)	100 (29.1)
Total	169 (49.1)	85 (24.7)	90 (26.2)	344

	pN0	pN+	Total
cN0	44 (12.8)	33 (9.6)	77 (22.4)
cN+	125 (36.3)	142 (41.3)	267 (77.6)
Total	169 (49.1)	175 (50.9)	344

	pEMVI present	pEMVI absent	Total
cEMVI present	110 (32.6)	107 (31.8)	217 (64.4)
cEMVI absent	45 (13.4)	75 (22.3)	120 (35.6)
Total	155 (46.0)	182 (54.0)	337

Green shading represents radiological and pathological concordance which was used to calculate overall agreement. Numbers in brackets represent staging combination as a percentage of the overall cohort.

Abbreviations: c = radiological; EMVI = extramural venous invasion; N = lymph node stage/status; p = pathological; T = primary tumour stage.

**Table 2.** Radiological staging performance according to MMR status and primary tumour side.

		Overall agreement, (% [95% CI])	Sensitivity, (% [95% CI])	Specificity, (% [95% CI])	PPV, (% [95% CI])	NPV, (% [95% CI])
All (*n* = 354)	T stage/status^a^	220/349 (63.0 [57.7-68.1])			329/348 (94.5 [91.6-96.7])	
	N status^b^	186/344 (54.1 [48.6-59.4])	142/175 (81.1 [74.5-86.6])	44/169 (26.0 [19.6-33.3])	142/267 (53.2 [47.0-59.3])	44/77 (57.1 [45.4-68.4])
	EMVI status	185/337 (54.9 [49.4-60.3])	110/155 (71.0 [63.1-78.0])	75/182 (41.2 [34.0-48.7])	110/217 (50.7 [43.8-57.5])	75/120 (62.5 [53.2-71.2])
pMMR^c^^,^^d^ (*n* = 248)	T stage/status^a^	153/247 (61.9 [55.6-68.0])			230/246 (93.5 [90.5-96.4])	
	N status^b^	136/245 (55.5 [49.0-61.8])	106/133 (79.7 [71.9-86.2])	30/112 (26.8 [18.9-36.0])	106/188 (56.4 [49.0-63.6])	30/57 (52.6 [39.0-66.0])
	EMVI status	140/242 (57.9 [51.4-64.2])	83/117 (70.9 [61.8-79.0])	57/125 (45.6 [36.7-54.7])	83/151 (55.0 [46.7-63.1])	57/91 (62.6 [51.9-72.6])
dMMR^c^^,^^d^ (*n* = 68)	T stage/status^a^	42/67 (62.7 [50.0-74.2])			65/67 (97.0 [89.6-99.6])	
	N status^b^	36/68 (52.9 [40.5-65.2])	25/28 (89.3 [71.8-97.7])	11/40 (27.5 [14.6-43.9])	25/54 (46.3 [32.6-60.4])	11/14 (78.6 [49.2-95.3])
	EMVI status	30/65 (46.2 [33.7-59.0])	19/27 (70.4 [49.8-86.2])	11/38 (28.9 [15.4-45.9])	19/46 (41.3 [27.0-56.8])	11/19 (57.9 [33.5-79.7])
Right side^e^^,^^f^ (*n* = 175)	T stage/status^a^	103/173 (59.5 [51.8-66.9])			166/172 (96.5 [92.5-98.7])	
	N status^b^	89/171 (52.0 [44.3-59.7])	74/86 (86.0 [76.9-92.6])	15/85 (17.6 [10.2-27.4])	74/144 (51.4 [42.9-59.8])	15/27 (55.6 [35.3-74.5])
	EMVI status	90/166 (54.2 [46.3-61.2])	52/70 (74.3 [62.4-84.0])	38/96 (39.6 [29.7-50.1])	52/110 (47.3 [37.7-57.0])	38/56 (67.9 [54.0-79.7])
Left side^f^^,^^g^ (*n* = 175)	T stage/status^a^	117/174 (67.2 [59.7-74.2])			161/174 (92.5 [87.8-96.1])	
	N status^b^	96/170 (56.5 [48.7-64.0])	68/88 (77.3 [67.1-85.5])	28/82 (34.1 [24.0-45.4])	68/122 (55.7 [46.5-64.7])	28/48 (58.3 [43.2-72.4])
	EMVI status	93/168 (55.4 [47.5-63.0])	58/84 (69.0 [58.0-78.7])	35/84 (41.7 [31.0-52.9])	58/107 (54.2 [44.3-63.9])	35/61 (57.4 [44.1-70.0])

Abbreviations: dMMR = mismatch repair deficient; EMVI = extramural venous invasion; MMR = mismatch repair; NPV = negative predictive value; pMMR = mismatch repair proficient; PPV = positive predictive value.

a Individual T stage used for overall agreement. Categorized as T1-2 or T3-4 for calculation of PPV.

b N stage categorized as N0 or N+.

c According to immunohistochemistry.

d MMR status unknown for 38 participants.

e Tumours proximal to the splenic flexure.

f Tumour side unknown for 4 participants.

g Tumours at or distal to the splenic flexure.

### N staging

Both radiological and pathological N stage data were available in 97.2% (344/354) of participants (Figure 1).

**Figure 4.**
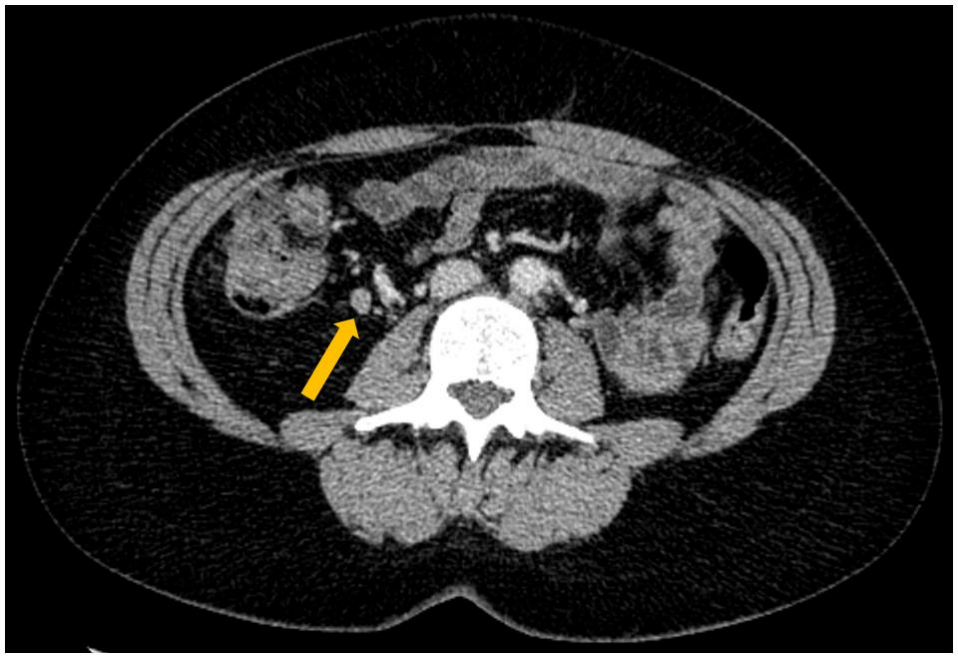
Example of incorrect N staging. Baseline axial image of a participant in the straight-to-surgery arm with an ascending colon tumour. This case was radiologically staged cN2 and pathologically staged pN0. Yellow arrow indicates a regional lymph node suspicious for malignant involvement.

Overall N stage agreement was 39.8% (137/344) (Table 1). Pathological N0, N1, and N2 staging were correctly predicted in 26.0% (44/169), 60.0% (51/85), and 46.7% (42/90) of participants, respectively (Table 1; Figure 4). The overall agreement, sensitivity, specificity, PPV, and NPV of CT for predicting N status were 54.1% (186/344), 81.1% (142/175), 26.0% (44/169), 53.2% (142/267), and 57.1% (44/77), respectively (Tables 1 and 2; Figure 3).

### EMVI status

Both radiological and pathological EMVI status data were available in 95.2% (337/354) of participants (Figure 1).

The overall agreement, sensitivity, specificity, PPV, and NPV of CT for predicting EMVI status were 54.9% (185/337), 71.0% (110/155), 41.2% (75/182), 50.7% (110/217), and 62.5% (75/120), respectively (Tables 1 and 2; Figure 3).

### Impact of MMR status and primary tumour side

Table 2 summarizes the impact of MMR status and primary tumour side on staging concordance. Of the 248 participants with MMR proficient (pMMR) tumours, pathological T stage was T1 in a single participant, T2 in 16 participants, T3 in 159 participants, and T4 in 72 participants; 133 participants were pathologically staged N+ (N1 = 66 and N2 = 67) and 118 participants were found to have EMVI (Table S4). For the 68 participants with dMMR tumours, pathological T stage was T2 in 2 participants, T3 in 42 participants, and T4 in 24 participants; no participants had a T1 tumour; 28 participants were pathologically staged N+ (N1 = 11 and N2 = 17) and 27 were found to have EMVI (Table S5).

A total of 175 participants had a right-sided tumour, where pathological T stage was T2 in 6 participants, T3 in 109 participants, and T4 in 58 participants; no participants had a T1 tumour; 86 participants were pathologically staged N+ (N1 = 41 and N2 = 45) and 70 had EMVI (Table S6). Likewise, 175 participants had a left-sided tumour, where pathological T stage was T1 in a single participant, T2 in 12 participants, T3 in 114 participants, and T4 in 47 participants. Eighty-nine participants were pathologically staged N+ (N1 = 45 and N2 = 44) and 85 had EMVI (Table S7).

Numerical differences in staging correlation were observed according to MMR status and tumour side but overlapping CIs indicate that these may not be significant (Table 2). While dMMR tumours are more common in the right colon and therefore represent a potential confounder, similar differences were seen according to tumour side when limited to just pMMR tumours: 57.9% (66/114) vs. 67.5% (112/166) for T stage agreement; 14.0% (7/50) vs. 32.1% (25/78) for N status specificity; and 72.5% (29/40) vs. 57.6% (34/59) for EMVI NPV in those with right, compared to left, sided tumours (Tables S8 and S9).

## Discussion

FOxTROT was the first phase III, randomized-controlled trial to show NAC significantly reduces the risk of recurrence in patients with locally advanced CC. Our analysis of prospective radiological staging by trained radiologists shows a combined T3-4 CT staging group is a reliable biomarker for neoadjuvant therapy patient selection with minimal overstaging of T1-2 tumours against final pathology. However, we also determined that CT cannot reliably select patients in the higher-risk T3 (≥5 mm extramural extension)-T4 or T4 alone groups and reaffirmed the limitations of CT for predicting N stage and EMVI status.^4^^,^^5^^,^^10^^,^^23^ In our opinion, these features, which are more challenging to identify, should be avoided or used with caution for patient selection in other neoadjuvant trials. These data underscore the role of simplified radiological T staging (T3-4) as the key biomarker for patient selection, bringing CC in line with other gastrointestinal malignancies.^24^^,^^25^

The PPV of CT for predicting T3-4 tumours was 94.5%, which is similar to other studies (90%-92%).^14^^,^^15^^,^^20^ At least 1 mm extramural extension on CT was required for trial eligibility because microscopic extension beyond the muscularis propria is unlikely to be visible within the limitations of CT contrast and spatial resolution. While the assessment of minimal tumour extension on CT is challenging and represents an area of uncertainty with regards to emerging neoadjuvant therapies, it is important to acknowledge that measurements on CT and pathology are not necessarily comparable; CT offers an assessment of the whole tumour *in vivo*, whereas pathological assessment may alter as a consequence of fixation and involves evaluation of select slices, rather than the whole specimen. Nevertheless, tumours with <1 mm extension are not a focus for neoadjuvant therapy, since they confer a lower-risk of disease recurrence and the benefit of systemic therapy is likely to be negligible.^26^ Limiting T3 tumours to only those with ≥5 mm extramural extension substantially lowered the PPV, suggesting this higher-risk group cannot be as reliably identified by CT. In addition, and consistent with our pilot data, CT struggled to differentiate T3 and T4 tumours, which may reflect the difficulty posed by T4 tumours with microscopic extension through the peritoneum.^23^

Only 20 (5.6%) participants had T1-2 tumours that were radiologically overstaged. However, other high-risk features (N+ and/or EMVI) were present in 8 of these participants. Considering established approaches to adjuvant treatment, it is possible that such patients may also benefit from NAC.^3^

Whilst we could only calculate the PPV for T status, other studies consistently report limited NPV (38%-51%).^4^^,^^5^^,^^11^^,^^13–15^^,^^20^^,^^27^^,^^28^ This may lead to understaging and reduced NAC provision, but these patients would still undergo surgery with or without adjuvant chemotherapy, which remains an effective treatment option.^3^

T stage agreement was numerically greater in left-sided than right-sided tumours (67.2% vs. 59.5%, respectively) in both the whole STS arm and just those with pMMR tumours. To the best of our knowledge, this finding is not described elsewhere, may reflect the biological, morphological, and anatomical differences between the right and left colon, and should be a subject for further research.^29^

The limitations of radiological N staging due to micrometastases, small vessel EMVI, and reactive lymphadenopathy are well-recognized. N stage agreement was present in only 39.8% of participants, increasing to 54.1% when categorized as N status. The sensitivity and specificity of CT for predicting N status contrasts with wider literature (81.1% and 26.0% vs. 55%-71% and 66%-78%, respectively).^4^^,^^5^^,^^14^^,^^15^^,^^20^ This distinction may reflect the inclusion of exclusively advanced tumours, which may bias reporting to incorrectly predict lymph node metastases.

A numerically lower PPV and specificity for predicting N status in dMMR (vs. pMMR) and right (vs. left)-sided tumours, respectively, are consistent with a Danish population-level study.^20^ Lower prevalence of lymph node metastases and larger size of reactive nodes in dMMR than pMMR CC may explain the higher observed NPV.^30^ These data further reinforce the need to consider MMR status when radiologically staging CC.^20^^,^^21^^,^^31^^,^^32^

While MRI assessment of EMVI in rectal cancer is well-established, the evidence for CT-detected EMVI in CC is limited.^33^ In this analysis, the sensitivity and specificity of CT for detecting EMVI is disappointing and differs from other reports (71.0% and 41.2% vs. 35%-63% and 67%-94%, respectively).^10^^,^^13^^,^^34–36^ Again, we suspect that this may reflect the inclusion of exclusively advanced disease, where greater extramural extension, perineural or lymphatic invasion, and peritumoural inflammation may be mistaken for EMVI on CT. Furthermore, CT assessment of EMVI may be limited by the involvement of small vessels which are unlikely to be visible. The inflammatory nature of dMMR CC may also explain particularly poor agreement in this subgroup, where subtle peritumoural inflammation could be misinterpreted as EMVI.^30^

Macroscopic and microscopic pathological examination has long been established as the gold standard for guiding decisions on chemotherapy and predicting prognosis in CC.^3^ In the era of precision oncology, the adoption of routine molecular biomarkers, such as MMR status, complements pathological staging to enhance the selection of systemic therapies. It is clear that complete matching of radiological and pathological TNM staging is imprecise and appears to be a futile endeavour. However, neoadjuvant treatment requires *in vivo* assessment of colon tumours. While our data show that CT is able to accurately select patients with T3 tumours and above, the limited prediction of other features indicates that we must move beyond the paradigm of “pathology matching” and towards “radiological phenotyping.”

Current radiology staging assessments are confined to the TNM system, which neglects features that may be effective radiological biomarkers. With the rapid expansion of neoadjuvant trials in CC, there is a need to optimize and harmonize radiological assessment, while identifying independent imaging biomarkers and those which contribute to a broader multimodal approach.^37^^,^^38^ Recent studies have identified additional biomarkers which support TNM staging, including a specific vascular pattern to differentiate T3 and T4a tumours (“small arteriole sign”), and systemic inflammatory markers, lymph node irregularity, and lymph node heterogeneity to predict N status.^21^^,^^39–41^ However, these studies remain focused on TNM staging and the prediction of pathology over clinical outcomes, and are limited by smaller size, retrospective design, and a lack of validation.

Some studies have explored the performance of MRI and PET-CT, aiming to overcome the limitations of CT lymph node and EMVI assessment. Neither demonstrate superior performance for N staging, and while there may be some improvement in EMVI detection with MRI, this is not conclusive.^42^ Furthermore, these data often comprise expert reviews which may not reflect real-world performance.^10^^,^^13^^,^^43^ Increasingly, research is utilizing radiomics and artificial intelligence applications to support radiological staging, but this remains experimental. CT, PET-CT, and MRI radiomics have all shown promise for predicting N stage, which may have clinical utility for patient selection in the era of neoadjuvant therapies. Such approaches may also deliver novel imaging biomarkers to improve patient risk assessment and further support treatment stratification.^44^^,^^45^ The value of radiological response to NAC has not been comprehensively evaluated and represents a new avenue for imaging research.

Radiologists and cancer specialists must recognize the importance of training to deliver accurate CC staging in routine practice.^46^^,^^47^ This trial successfully delivered a bespoke radiology training programme to local radiologists, resulting in strong performance for T stage assessment. Other benefits of this training may become clear once radiology-pathology correlation data is published for other neoadjuvant trials.^19^ Analyses of trial datasets should be used to identify areas of unmet need to inform the development of training resources. We identified lymph node and EMVI assessment as clear weaknesses; however, in the context of such poor performance, it is unlikely that further training alone will produce substantial improvements. As novel features and biomarkers are developed and validated, training will be critical to ensure wider radiological practice advances efficiently. We recommend that proformas and formal training are considered a key component of any future trials in this setting to ensure consistency and generalizability. Template reporting of CC CT may also support the description and standardization of key imaging biomarkers in routine, multidisciplinary practice, as is the case in rectal cancer.^48^

This analysis is limited by the inclusion of exclusively advanced disease, which may influence several aspects of staging performance and affect the generalizability of findings to radiological evaluation in the wider CC population. The results may also be limited by differences between TNM version 5 and the contemporary version 8. For example, tumour deposits <3 mm were previously considered discontinuous tumour extension as part of T staging, as opposed to N staging in version 8 (N1c in the absence of lymph node metastases).^49^ The exclusion of patients with <1 mm extramural extension on CT may also enhance performance by excluding challenging, borderline cases. Interobserver variability could not be assessed due to the design of the trial. However, the delivery of bespoke radiological training and use of established radiological features ensure a degree of standardization. While the lack of central review will introduce heterogeneity to the data and could be considered a limitation, we believe that focusing on staging performance in real-world, multicentre practice, which delivered a positive trial, to be major strength. This evaluation of prospective, local staging performance in a large, international trial indicates broader applicability of this approach in routine clinical practice.

We have shown that CT reliably identifies a combined T3-4 staging group with minimal overstaging versus final pathology. However, limited performance was seen for assessing tumour extension, individual T stage, N stage, or EMVI status. We propose that T3-4 staging on CT should be considered the standard biomarker to select patients with CC for neoadjuvant therapies. Radiological phenotyping through the identification of additional imaging biomarkers is a priority to effectively predict benefit from neoadjuvant therapies to build multimodal approaches for patient selection.

## Supplementary Material

tqaf217_Supplementary_Data
