# Supplementary material for: CT staging performance in an international trial of neoadjuvant chemotherapy for locally advanced colon cancer
Source: Br J Radiol. 2025 Aug 23;98(1176):2175–83. doi: 10.1093/bjr/tqaf217 (PMC12810875; doi:10.1093/bjr/tqaf217)
Supplement: tqaf217_Supplementary_Data [file tqaf217_supplementary_data.docx]

**Supplementary Data**

**Table S1. Baseline Clinical and Staging Characteristics**

|  | NAC (n = 698) | STS (n = 354) | P-value ^c^ |
| --- | --- | --- | --- |
| Age, years (range; SD) |  |  |  |
| Mean | 63.0 (27 – 82; 10) | 63.2 (31 – 83; 9) | 0.69 |
| Sex, n (%) |  |  |  |
| Male | 447 (64.0%) | 225 (63.6%) | 0.88 |
| Female | 251 (36.0%) | 129 (36.4%) |  |
| Tumour location, n (%) |  |  |  |
| Caecum | 119 (17.0%) | 63 (17.8%) | 0.96 |
| Ascending colon | 124 (17.8%) | 64 (18.1%) |  |
| Hepatic flexure | 41 (5.9%) | 22 (6.2%) |  |
| Transverse colon | 49 (7.0%) | 26 (6.2%) |  |
| Splenic flexure | 27 (3.9%) | 9 (2.5%) |  |
| Descending colon | 36 (5.2%) | 22 (6.2%) |  |
| Sigmoid | 250 (35.8%) | 122 (34.5%) |  |
| Rectosigmoid junction | 46 (6.6%) | 22 (6.2%) |  |
| Unknown | 6 (0.9%) | 4 (1.1%) |  |
| Tumour side, n (%) |  |  |  |
| Right | 333 (47.7%) | 175 (49.4%) | 0.57 |
| Left | 359 (51.4%) | 175 (49.4%) |  |
| Unknown | 6 (0.9%) | 4 (1.1%) |  |
| MMR status, n (%) ^a^ |  |  |  |
| Proficient | 481 (68.9%) | 248 (70.1%) | 0.49 |
| Deficient | 117 (16.8%) | 68 (19.2%) |  |
| Unknown | 100 (14.3%) | 38 (10.7%) |  |
| Radiological T stage, n (%) ^b^ |  |  |  |
| T2 | 1 (0.1%) | 1 (0.3%) | 0.42 |
| T3 | 523 (74.9%) | 256 (72.3%) |  |
| T4 | 169 (24.2%) | 95 (26.8%) |  |
| Unknown | 5 (0.7%) | 2 (0.6%) |  |
| Radiological N stage, n (%) ^b^ |  |  |  |
| N0 | 169 (24.2%) | 81 (22.9%) | 0.88 |
| N1 | 328 (47.0%) | 169 (47.7%) |  |
| N2 | 196 (28.1%) | 102 (28.8%) |  |
| Unknown | 5 (0.7%) | 2 (0.6%) |  |
| Radiological EMVI, n (%) |  |  |  |
| Present | 408 (58.5%) | 226 (63.8%) | 0.11 |
| Absent | 280 (40.1%) | 125 (35.3%) |  |
| Unknown | 10 (1.4%) | 3 (0.9%) |  |
| Pathological T stage, n (%) ^b^ |  |  |  |
| T1 | 10 (1.4%) | 1 (0.3%) | 0.0004 |
| T2 | 70 (10.0%) | 19 (5.4%) |  |
| T3 | 436 (62.5%) | 224 (63.3%) |  |
| T4 | 142 (20.3%) | 107 (30.2%) |  |
| Unknown | 40 (5.7%) | 3 (0.9%) |  |
| Pathological N stage, n (%) ^b^ |  |  |  |
| N0 | 406 (58.2%) | 169 (47.7%) | 0.0001 |
| N1 | 173 (24.8%) | 87 (24.6%) |  |
| N2 | 104 (14.9%) | 90 (25.4%) |  |
| Unknown | 15 (2.1%) | 8 (2.3%) |  |
| Pathological EMVI, n (%) |  |  |  |
| Present | 221 (31.7%) | 156 (44.1%) | 0.00004 |
| Absent | 456 (65.3%) | 184 (52.0%) |  |
| Unknown | 21 (3.0%) | 14 (4.0%) |  |
| EMVI = extramural venous invasion; MMR = mismatch repair; N = lymph node stage; NAC = neoadjuvant chemotherapy; SD = standard deviation; STS = straight-to-surgery; T = primary tumour stage.  ^a^ Tested using immunohistochemistry.  ^b^ According to TNM version 5.  ^c^ Pearson Chi-squared or Fisher’s exact test. Tests exclude unknowns. | | | |

**Table S2. Differences Between MDT and Lead Radiologist Data for Whole Study Population**

|  | **Lead Radiologist Review** | | | | | | | | | | | | | | | | | | | | | | | | |
| --- | --- | --- | --- | --- | --- | --- | --- | --- | --- | --- | --- | --- | --- | --- | --- | --- | --- | --- | --- | --- | --- | --- | --- | --- | --- |
| **MDT Review** | **Primary Tumour Site** | | | | | | | | | | | | | | | | | | | | | | | | |
|  |  | Caecum | | | Ascending colon | | Hepatic flexure | | Transverse colon | | | Splenic flexure | | Descending colon | | | Sigmoid | | | Rectosigmoid | | | Other/ missing | | TOTAL |
|  | Caecum | 168 | | | 9 | | 0 | | 0 | | | 0 | | 1 | | | 2 | | | 0 | | | 2 | | 182 |
|  | Ascending colon | 10 | | | 170 | | 1 | | 0 | | | 0 | | 1 | | | 0 | | | 0 | | | 3 | | 185 |
|  | Hepatic flexure | 0 | | | 7 | | 55 | | 1 | | | 1 | | 0 | | | 0 | | | 0 | | | 2 | | 66 |
|  | Transverse colon | 0 | | | 2 | | 7 | | 71 | | | 0 | | 1 | | | 0 | | | 0 | | | 0 | | 81 |
|  | Splenic flexure | 0 | | | 0 | | 0 | | 3 | | | 29 | | 0 | | | 2 | | | 0 | | | 0 | | 34 |
|  | Descending colon | 0 | | | 0 | | 0 | | 0 | | | 1 | | 52 | | | 1 | | | 0 | | | 1 | | 55 |
|  | Sigmoid | 4 | | | 0 | | 0 | | 0 | | | 5 | | 3 | | | 350 | | | 8 | | | 1 | | 371 |
|  | Rectosigmoid | 0 | | | 0 | | 0 | | 0 | | | 0 | | 0 | | | 17 | | | 60 | | | 1 | | 78 |
|  | Other/ missing | 0 | | | 0 | | 0 | | 0 | | | 0 | | 0 | | | 0 | | | 0 | | | 0 | | 0 |
|  | TOTAL | 182 | | | 188 | | 63 | | 75 | | | 36 | | 58 | | | 372 | | | 68 | | | 10 | | 1052 |
|  | **T Stage** | | | | | | | | | | | | | | | | | | | | | | | | |
|  |  | | T1 | | | T2 | | | | | T3 | | | | T4 | | | | Unknown | | | | | TOTAL | |
|  | T1 | | 0 | | | 0 | | | | | 0 | | | | 0 | | | | 0 | | | | | 0 | |
|  | T2 | | 0 | | | 0 | | | | | 0 | | | | 0 | | | | 0 | | | | | 0 | |
|  | T3 | | 0 | | | 2 | | | | | 743 | | | | 35 | | | | 4 | | | | | 784 | |
|  | T4 | | 0 | | | 0 | | | | | 36 | | | | 229 | | | | 3 | | | | | 268 | |
|  | Unknown | | 0 | | | 0 | | | | | 0 | | | | 0 | | | | 0 | | | | | 0 | |
|  | TOTAL | | 0 | | | 2 | | | | | 779 | | | | 264 | | | | 7 | | | | | 1052 | |
|  | **Degree of Extramural Extension** | | | | | | | | | | | | | | | | | | | | | | | | |
|  |  | | | | <5mm | | | | | ≥5mm | | | | | | Unknown | | | | | TOTAL | | | | |
|  | <5mm | | | | 257 | | | | | 37 | | | | | | 2 | | | | | 296 | | | | |
|  | ≥5mm | | | | 33 | | | | | 722 | | | | | | 1 | | | | | 756 | | | | |
|  | Unknown | | | | 0 | | | | | 0 | | | | | | 0 | | | | | 0 | | | | |
|  | TOTAL | | | | 290 | | | | | 759 | | | | | | 3 | | | | | 1052 | | | | |
|  | **N Stage** | | | | | | | | | | | | | | | | | | | | | | | | |
|  |  | | | N0 | | | | N1 | | | | | N2 | | | | | Unknown | | | | TOTAL | | | |
|  | N0 | | | 210 | | | | 26 | | | | | 5 | | | | | 2 | | | | 243 | | | |
|  | N1 | | | 30 | | | | 431 | | | | | 42 | | | | | 2 | | | | 505 | | | |
|  | N2 | | | 5 | | | | 31 | | | | | 249 | | | | | 2 | | | | 287 | | | |
|  | Unknown | | | 5 | | | | 9 | | | | | 2 | | | | | 1 | | | | 17 | | | |
|  | TOTAL | | | 250 | | | | 497 | | | | | 298 | | | | | 7 | | | | 1052 | | | |

**Table S3. T Stage Agreement Including T3<5mm vs. T3 ≥5mm**

|  | pT1 | pT2 | pT3 <5mm | pT3 ≥5mm | pT4 | Unknown | Total |
| --- | --- | --- | --- | --- | --- | --- | --- |
| cT1 | 0 | 0 | 0 | 0 | 0 | 0 | 0 |
| cT2 | 0 | 0 | 0 | 0 | 1 | 0 | 1 |
| cT3 <5mm | 0 | 8 | 40 | 15 | 20 | 4 | 87 |
| cT3 ≥5mm | 1 | 7 | 48 | 61 | 42 | 10 | 169 |
| cT4 | 0 | 3 | 20 | 26 | 44 | 2 | 95 |
| Unknown | 0 | 1 | 0 | 1 | 0 | 0 | 2 |
| Total | 1 | 19 | 108 | 103 | 107 | 16 | 354 |

**Table S4. Agreement for pMMR Tumours**

|  | pT1 | | | pT2 | | | pT3 | | pT4 | | | Unknown | | | Total |
| --- | --- | --- | --- | --- | --- | --- | --- | --- | --- | --- | --- | --- | --- | --- | --- |
| cT1 | 0 | | | 0 | | | 0 | | 0 | | | 0 | | | 0 |
| cT2 | 0 | | | 0 | | | 0 | | 1 | | | 0 | | | 1 |
| cT3 | 1 | | | 12 | | | 126 | | 44 | | | 0 | | | 183 |
| cT4 | 0 | | | 3 | | | 33 | | 27 | | | 0 | | | 63 |
| Unknown | 0 | | | 1 | | | 0 | | 0 | | | 0 | | | 1 |
| Total | 1 | | | 16 | | | 159 | | 72 | | | 0 | | | 248 |
|  | | | | | | | | | | | | | | | |
|  | | | pT1-2 | | | pT3-4 | | | | Unknown | | | Total | | |
| cT1-2 | | | 0 | | | 1 | | | | 0 | | | 1 | | |
| cT3-4 | | | 16 | | | 230 | | | | 0 | | | 246 | | |
| Unknown | | | 1 | | | 0 | | | | 0 | | | 1 | | |
| Total | | | 17 | | | 231 | | | | 0 | | | 248 | | |
|  | | | | | | | | | | | | | | | |
|  | | pN0 | | | pN1 | | | pN2 | | | Unknown | | | Total | |
| cN0 | | 30 | | | 15 | | | 12 | | | 0 | | | 57 | |
| cN1 | | 54 | | | 39 | | | 24 | | | 1 | | | 118 | |
| cN2 | | 28 | | | 12 | | | 31 | | | 0 | | | 71 | |
| Unknown | | 0 | | | 2 | | | 0 | | | 0 | | | 2 | |
| Total | | 112 | | | 68 | | | 67 | | | 1 | | | 248 | |
|  | | | | | | | | | | | | | | | |
|  | | | pEMVI present | | | pEMVI absent | | | | Unknown | | | Total | | |
| cEMVI present | | | 83 | | | 68 | | | | 3 | | | 154 | | |
| cEMVI absent | | | 34 | | | 57 | | | | 0 | | | 91 | | |
| Unknown | | | 1 | | | 2 | | | | 0 | | | 3 | | |
| Total | | | 118 | | | 127 | | | | 3 | | | 248 | | |

**Table S5. Agreement for dMMR Tumours**

|  | pT1 | | | pT2 | | | pT3 | | pT4 | | | Unknown | | | Total |
| --- | --- | --- | --- | --- | --- | --- | --- | --- | --- | --- | --- | --- | --- | --- | --- |
| cT1 | 0 | | | 0 | | | 0 | | 0 | | | 0 | | | 0 |
| cT2 | 0 | | | 0 | | | 0 | | 0 | | | 0 | | | 0 |
| cT3 | 0 | | | 2 | | | 30 | | 12 | | | 0 | | | 44 |
| cT4 | 0 | | | 0 | | | 11 | | 12 | | | 0 | | | 23 |
| Unknown | 0 | | | 0 | | | 1 | | 0 | | | 0 | | | 1 |
| Total | 0 | | | 2 | | | 42 | | 24 | | | 0 | | | 68 |
|  | | | | | | | | | | | | | | | |
|  | | | pT1-2 | | | pT3-4 | | | | Unknown | | | Total | | |
| cT1-2 | | | 0 | | | 0 | | | | 0 | | | 0 | | |
| cT3-4 | | | 2 | | | 65 | | | | 0 | | | 67 | | |
| Unknown | | | 0 | | | 1 | | | | 0 | | | 1 | | |
| Total | | | 2 | | | 66 | | | | 0 | | | 68 | | |
|  | | | | | | | | | | | | | | | |
|  | | pN0 | | | pN1 | | | pN2 | | | Unknown | | | Total | |
| cN0 | | 11 | | | 2 | | | 1 | | | 0 | | | 14 | |
| cN1 | | 17 | | | 8 | | | 8 | | | 0 | | | 33 | |
| cN2 | | 12 | | | 1 | | | 8 | | | 0 | | | 21 | |
| Unknown | | 0 | | | 0 | | | 0 | | | 0 | | | 0 | |
| Total | | 40 | | | 11 | | | 17 | | | 0 | | | 68 | |
|  | | | | | | | | | | | | | | | |
|  | | | pEMVI present | | | pEMVI absent | | | | Unknown | | | Total | | |
| cEMVI present | | | 19 | | | 27 | | | | 2 | | | 48 | | |
| cEMVI absent | | | 8 | | | 11 | | | | 1 | | | 20 | | |
| Unknown | | | 0 | | | 0 | | | | 0 | | | 0 | | |
| Total | | | 27 | | | 38 | | | | 3 | | | 68 | | |

**Table S6. Agreement for Right-Sided Tumours**

|  | pT1 | | | pT2 | | | pT3 | | pT4 | | | Unknown | | | Total |
| --- | --- | --- | --- | --- | --- | --- | --- | --- | --- | --- | --- | --- | --- | --- | --- |
| cT1 | 0 | | | 0 | | | 0 | | 0 | | | 0 | | | 0 |
| cT2 | 0 | | | 0 | | | 0 | | 1 | | | 0 | | | 1 |
| cT3 | 0 | | | 6 | | | 78 | | 32 | | | 1 | | | 117 |
| cT4 | 0 | | | 0 | | | 31 | | 25 | | | 1 | | | 57 |
| Unknown | 0 | | | 0 | | | 0 | | 0 | | | 0 | | | 0 |
| Total | 0 | | | 6 | | | 109 | | 58 | | | 2 | | | 175 |
|  | | | | | | | | | | | | | | | |
|  | | | pT1-2 | | | pT3-4 | | | | Unknown | | | Total | | |
| cT1-2 | | | 0 | | | 1 | | | | 0 | | | 1 | | |
| cT3-4 | | | 6 | | | 166 | | | | 2 | | | 174 | | |
| Unknown | | | 0 | | | 0 | | | | 0 | | | 0 | | |
| Total | | | 6 | | | 167 | | | | 2 | | | 175 | | |
|  | | | | | | | | | | | | | | | |
|  | | pN0 | | | pN1 | | | pN2 | | | Unknown | | | Total | |
| cN0 | | 15 | | | 9 | | | 3 | | | 2 | | | 29 | |
| cN1 | | 41 | | | 25 | | | 19 | | | 2 | | | 87 | |
| cN2 | | 29 | | | 7 | | | 23 | | | 0 | | | 59 | |
| Unknown | | 0 | | | 0 | | | 0 | | | 0 | | | 0 | |
| Total | | 85 | | | 41 | | | 45 | | | 4 | | | 175 | |
|  | | | | | | | | | | | | | | | |
|  | | | pEMVI present | | | pEMVI absent | | | | Unknown | | | Total | | |
| cEMVI present | | | 52 | | | 58 | | | | 5 | | | 115 | | |
| cEMVI absent | | | 18 | | | 38 | | | | 3 | | | 59 | | |
| Unknown | | | 0 | | | 1 | | | | 0 | | | 1 | | |
| Total | | | 70 | | | 97 | | | | 8 | | | 175 | | |

**Table S7. Agreement for Left-Sided Tumours**

|  | pT1 | | | pT2 | | | pT3 | | pT4 | | | Unknown | | | Total |
| --- | --- | --- | --- | --- | --- | --- | --- | --- | --- | --- | --- | --- | --- | --- | --- |
| cT1 | 0 | | | 0 | | | 0 | | 0 | | | 0 | | | 0 |
| cT2 | 0 | | | 0 | | | 0 | | 0 | | | 0 | | | 0 |
| cT3 | 1 | | | 9 | | | 98 | | 28 | | | 1 | | | 137 |
| cT4 | 0 | | | 3 | | | 16 | | 19 | | | 0 | | | 38 |
| Unknown | 0 | | | 0 | | | 0 | | 0 | | | 0 | | | 0 |
| Total | 1 | | | 12 | | | 114 | | 47 | | | 1 | | | 175 |
|  | | | | | | | | | | | | | | | |
|  | | | pT1-2 | | | pT3-4 | | | | Unknown | | | Total | | |
| cT1-2 | | | 0 | | | 0 | | | | 0 | | | 0 | | |
| cT3-4 | | | 13 | | | 161 | | | | 1 | | | 175 | | |
| Unknown | | | 0 | | | 0 | | | | 0 | | | 0 | | |
| Total | | | 13 | | | 161 | | | | 1 | | | 175 | | |
|  | | | | | | | | | | | | | | | |
|  | | pN0 | | | pN1 | | | pN2 | | | Unknown | | | Total | |
| cN0 | | 28 | | | 10 | | | 10 | | | 2 | | | 50 | |
| cN1 | | 40 | | | 26 | | | 15 | | | 0 | | | 81 | |
| cN2 | | 14 | | | 8 | | | 19 | | | 2 | | | 43 | |
| Unknown | | 0 | | | 1 | | | 0 | | | 0 | | | 1 | |
| Total | | 82 | | | 45 | | | 44 | | | 4 | | | 175 | |
|  | | | | | | | | | | | | | | | |
|  | | | pEMVI present | | | pEMVI absent | | | | Unknown | | | Total | | |
| cEMVI present | | | 58 | | | 49 | | | | 4 | | | 111 | | |
| cEMVI absent | | | 26 | | | 35 | | | | 2 | | | 63 | | |
| Unknown | | | 1 | | | 0 | | | | 0 | | | 1 | | |
| Total | | | 85 | | | 84 | | | | 6 | | | 175 | | |

**Table S8. Agreement for pMMR and Right-Sided Tumours**

|  | pT1 | | | pT2 | | | pT3 | | pT4 | | | Unknown | | | Total |
| --- | --- | --- | --- | --- | --- | --- | --- | --- | --- | --- | --- | --- | --- | --- | --- |
| cT1 | 0 | | | 0 | | | 0 | | 0 | | | 0 | | | 0 |
| cT2 | 0 | | | 0 | | | 0 | | 1 | | | 0 | | | 1 |
| cT3 | 0 | | | 4 | | | 50 | | 21 | | | 1 | | | 76 |
| cT4 | 0 | | | 0 | | | 22 | | 16 | | | 1 | | | 39 |
| Unknown | 0 | | | 0 | | | 0 | | 0 | | | 0 | | | 0 |
| Total | 0 | | | 4 | | | 72 | | 38 | | | 2 | | | 116 |
|  | | | | | | | | | | | | | | | |
|  | | | pT1-2 | | | pT3-4 | | | | Unknown | | | Total | | |
| cT1-2 | | | 0 | | | 1 | | | | 0 | | | 1 | | |
| cT3-4 | | | 4 | | | 109 | | | | 2 | | | 115 | | |
| Unknown | | | 0 | | | 0 | | | | 0 | | | 0 | | |
| Total | | | 4 | | | 110 | | | | 2 | | | 116 | | |
|  | | | | | | | | | | | | | | | |
|  | | pN0 | | | pN1 | | | pN2 | | | Unknown | | | Total | |
| cN0 | | 7 | | | 8 | | | 2 | | | 2 | | | 19 | |
| cN1 | | 26 | | | 18 | | | 12 | | | 2 | | | 58 | |
| cN2 | | 17 | | | 6 | | | 16 | | | 0 | | | 39 | |
| Unknown | | 0 | | | 0 | | | 0 | | | 0 | | | 0 | |
| Total | | 50 | | | 32 | | | 30 | | | 4 | | | 116 | |
|  | | | | | | | | | | | | | | | |
|  | | | pEMVI present | | | pEMVI absent | | | | Unknown | | | Total | | |
| cEMVI present | | | 37 | | | 33 | | | | 3 | | | 73 | | |
| cEMVI absent | | | 11 | | | 29 | | | | 2 | | | 42 | | |
| Unknown | | | 0 | | | 1 | | | | 0 | | | 1 | | |
| Total | | | 48 | | | 63 | | | | 5 | | | 116 | | |

**Table S9. Agreement for pMMR and Left-Sided Tumours**

|  | pT1 | | | pT2 | | | pT3 | | pT4 | | | Unknown | | | Total |
| --- | --- | --- | --- | --- | --- | --- | --- | --- | --- | --- | --- | --- | --- | --- | --- |
| cT1 | 0 | | | 0 | | | 0 | | 0 | | | 0 | | | 0 |
| cT2 | 0 | | | 0 | | | 0 | | 0 | | | 0 | | | 0 |
| cT3 | 1 | | | 9 | | | 96 | | 27 | | | 1 | | | 134 |
| cT4 | 0 | | | 3 | | | 14 | | 16 | | | 0 | | | 33 |
| Unknown | 0 | | | 0 | | | 0 | | 0 | | | 0 | | | 0 |
| Total | 1 | | | 12 | | | 110 | | 43 | | | 1 | | | 167 |
|  | | | | | | | | | | | | | | | |
|  | | | pT1-2 | | | pT3-4 | | | | Unknown | | | Total | | |
| cT1-2 | | | 0 | | | 0 | | | | 0 | | | 0 | | |
| cT3-4 | | | 13 | | | 153 | | | | 1 | | | 167 | | |
| Unknown | | | 0 | | | 0 | | | | 0 | | | 0 | | |
| Total | | | 13 | | | 153 | | | | 1 | | | 167 | | |
|  | | | | | | | | | | | | | | | |
|  | | pN0 | | | pN1 | | | pN2 | | | Unknown | | | Total | |
| cN0 | | 25 | | | 9 | | | 10 | | | 2 | | | 46 | |
| cN1 | | 39 | | | 25 | | | 14 | | | 0 | | | 78 | |
| cN2 | | 14 | | | 8 | | | 18 | | | 2 | | | 42 | |
| Unknown | | 0 | | | 1 | | | 0 | | | 0 | | | 1 | |
| Total | | 78 | | | 43 | | | 42 | | | 4 | | | 167 | |
|  | | | | | | | | | | | | | | | |
|  | | | pEMVI present | | | pEMVI absent | | | | Unknown | | | Total | | |
| cEMVI present | | | 54 | | | 47 | | | | 4 | | | 105 | | |
| cEMVI absent | | | 25 | | | 34 | | | | 2 | | | 61 | | |
| Unknown | | | 1 | | | 0 | | | | 0 | | | 1 | | |
| Total | | | 80 | | | 81 | | | | 6 | | | 167 | | |
